# Acute Pancreatitis in the Transgender Population

**DOI:** 10.7759/cureus.16140

**Published:** 2021-07-03

**Authors:** Arslan Chaudhry, Rishitha Yelisetti, Christopher Millet, Christopher Biggiani, Shivanck Upadhyay

**Affiliations:** 1 Internal Medicine, St. Joseph’s Regional Medical Center, Paterson, USA; 2 Pulmonary and Critical Care Medicine, St. Joseph’s Regional Medical Center, Paterson, USA

**Keywords:** transgender, diabetes mellitus type 2, hormone replacement therapy, acute pancreatitis, hypertriglyceridemia-induced pancreatitis

## Abstract

Hypertriglyceridemia (HTG) is an uncommon but well-established etiology of acute pancreatitis (AP) leading to significant morbidity and mortality. Hormone replacement therapy in the transgender population is an underrecognized cause of elevated triglyceride (TG) levels and may put this group at a higher risk for severe pancreatitis. We present a case of AP in a genetically male patient receiving hormone therapy for female gender transformation.A 51-year-old with a past medical history of type 2 diabetes mellitus presented with severe epigastric abdominal pain associated with nonbilious, nonbloody vomiting and anorexia for two days. The patient was diagnosed with hypertriglyceridemia-induced acute pancreatitis (HTG-AP) in the setting of elevated lipase levels of 2,083 u/L and TGs of >5,000 mg/dL. In addition, a computerized tomography scan of the abdomen showed pancreatitis without evidence of necrosis. The patient was admitted to the medical intensive care unit for the management of AP in the setting of elevated TG levels. She was treated with intravenous fluids and an insulin drip. Her home medications including estradiol and Aldactone were held. Once the TG levels were reduced to <500 mg/dL, she was taken off the Insulin drip and transitioned to a subcutaneous insulin regimen along with gemfibrozil and omega-3 fatty acid over the next three days, and then discharged to home. HTG accounts for only about 7% of pancreatitis cases and increases in severity as TG levels increase. The clinical presentation of patients suffering from HTG-AP is similar to patients with AP from other etiologies and presents in a relatively younger population compared to AP from other causes. Treatment options for HTG-AP usually utilize insulin and heparin; however, plasma exchange and venovenous filtration may be used for severe cases of HTG-AP. The goal of treatment is to lower the TG levels. Physicians should be aware of such complications and should counsel patients while utilizing hormone replacement therapy, especially in patients with a prior family history of dyslipidemia.

## Introduction

Hypertriglyceridemia (HTG) is an uncommon but well-established etiology of acute pancreatitis (AP) leading to significant morbidity and mortality. The risk and severity of AP are suspected to increase with increasing levels of serum triglyceride (TG) levels [1]. It is crucial to identify HTG as the cause of pancreatitis and initiate an appropriate treatment plan. Hormone replacements in the transgender population are underrecognized as a cause of elevated TG levels and places this group at a higher risk for severe pancreatitis. Physicians should be aware of such complications and counsel patients while prescribing hormone replacements, especially in patients with a family history of dyslipidemia. Treatment of HTG depends on the severity of symptoms and the levels, with associated mortality. It may need aggressive interventions like plasma exchange (PLEX), especially in patients with severe pancreatitis, and resultant high mortality in these cases [1].

## Case presentation

**Figure 1:**
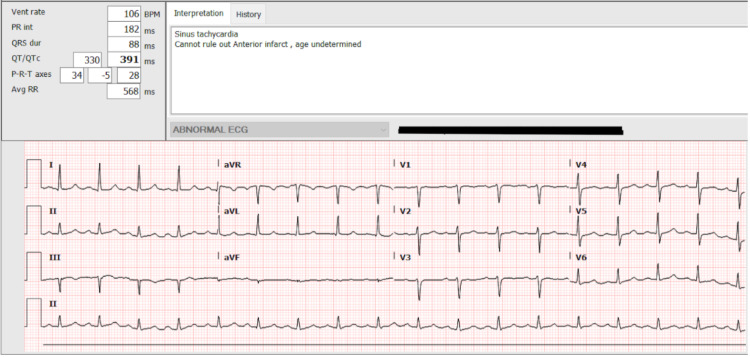
Electrocardiogram showing sinus tachycardia without any acute ST or T wave changes.

A 51-year-old genetic male (preferred to be addressed as she) with a past medical history of type 2 diabetes mellitus presented to the hospital with complaints of severe epigastric abdominal pain for two days. The pain was described as sudden in onset, sharp, and severe in the epigastric region. The patient rated her pain as severe in intensity, occasionally radiating to her back, and worsening in intensity while laying flat. She was unaware of any aggravating or alleviating factors. The pain was associated with episodes of nonbilious, nonbloody vomiting, and anorexia. She stated that she had a similar episode of pain about four weeks prior to the presentation which resolved within a few hours, thus the patient did not seek medical attention at that time. Home medications included Glipizide 5 mg PO QD, metformin 1,000 mg PO BID, estradiol 2 mg PO BID, and Aldactone 10 mg PO BID. At the time of presentation, her blood pressure was 145/100 mmHg, heart rate 86 beats per minute, respiratory rate 18 breaths per minute, and temperature 36.1°C. Her body mass index was 29.8 kg/m^2^. The patient was saturating 98% on room air. Pertinent positive findings on examination included significant epigastric tenderness and guarding without distension or rebound tenderness. Laboratory tests were significant for an elevated white blood cell count and an elevated lipase of 2,083 u/L. In addition, her TG was >5,000 mg/dL, HbA1c was 15.4%, and serum acetone was negative. Protein, albumin, alanine aminotransferase, aspartate aminotransferase, and alcohol levels were within the normal range. Furthermore, her Apache 2 score was 10 (7% estimated postoperative and 15% nonoperative mortality), Ranson score was 2 (1% predicted mortality), and neutrophil-to-lymphocyte ratio was 3.2 (a higher ratio indicating more severe pancreatitis). Table 1 lists the laboratory findings of the patient. Figure 1 shows the electrocardiogram findings of the patient which showed sinus tachycardia with no acute ST or T wave changes.

**Table 1:** Laboratory findings.

Lab parameters	Patient values	Normal range
Sodium (mEq/L)	122	135-145
Potassium (mEq/L)	4.8	3.5-5.0
Chloride (mEq/L)	90	98-107
Bicarbonate (mEq/L)	17	21-31
Glucose (mg/dL)	308	70-110
Calcium (mg/dL)	8.6	8.6-10.3
Phosphorus (mEq/L)	<1.0	2.5-5.0
Magnesium (mEq/L)	2.0	1.7-2.5
Blood urine nitrogen (mg/dL)	12	7-23
Serum creatinine (mg/dL)	0.9	0.6-1.3
Bilirubin total (mg/dL)	0.4	0.3-1.1
Protein total (g/dL)	6.3	6.4-8.4
Albumin (g/dL)	3.7	3.5-5.7
Alkaline phosphatase (U/L)	77	34-104
Aspartate aminotransferase (U/L)	25	13-39
Alanine aminotransferase (U/L)	7	7-52
Total cholesterol (mg/dL)	636	<199
Triglycerides (mg/dL)	>5,000	<149
Hemoglobin A1C (%)	15.4	4-6
Lipase (U/L)	2083	11-82
C-reactive protein (mg/L)	81.8	≤9.9
Lactic acid (mmol/L)	1.2	0.5-2.2
White blood cell count (×10^3^/mm^3^)	11.7	4.5-11.0
Hemoglobin (g/dL)	13.4	13.5-17.5
Hematocrit (%)	35.9	41.0-53.0
Mean corpuscular volume (fL)	86.9	80-100
Platelet (K/mm^3^)	428	140-440

**Figure 2:**
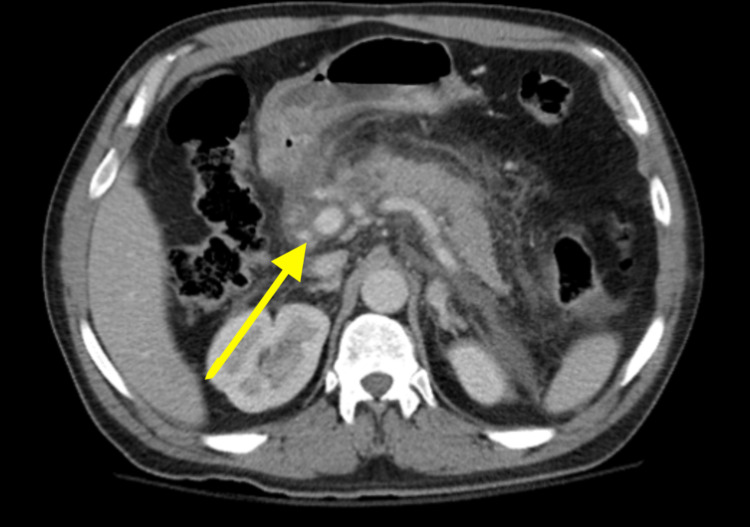
Computerized tomography scan of the abdomen showing pancreatitis without evidence of necrosis.

Limited ultrasound of the right upper abdomen was performed which showed fatty liver, adenomyomatosis in the gallbladder, and prominent pancreas. A computerized tomography scan of the abdomen revealed pancreatitis without any evidence of necrosis (Figure 2).

**Figure 3:**
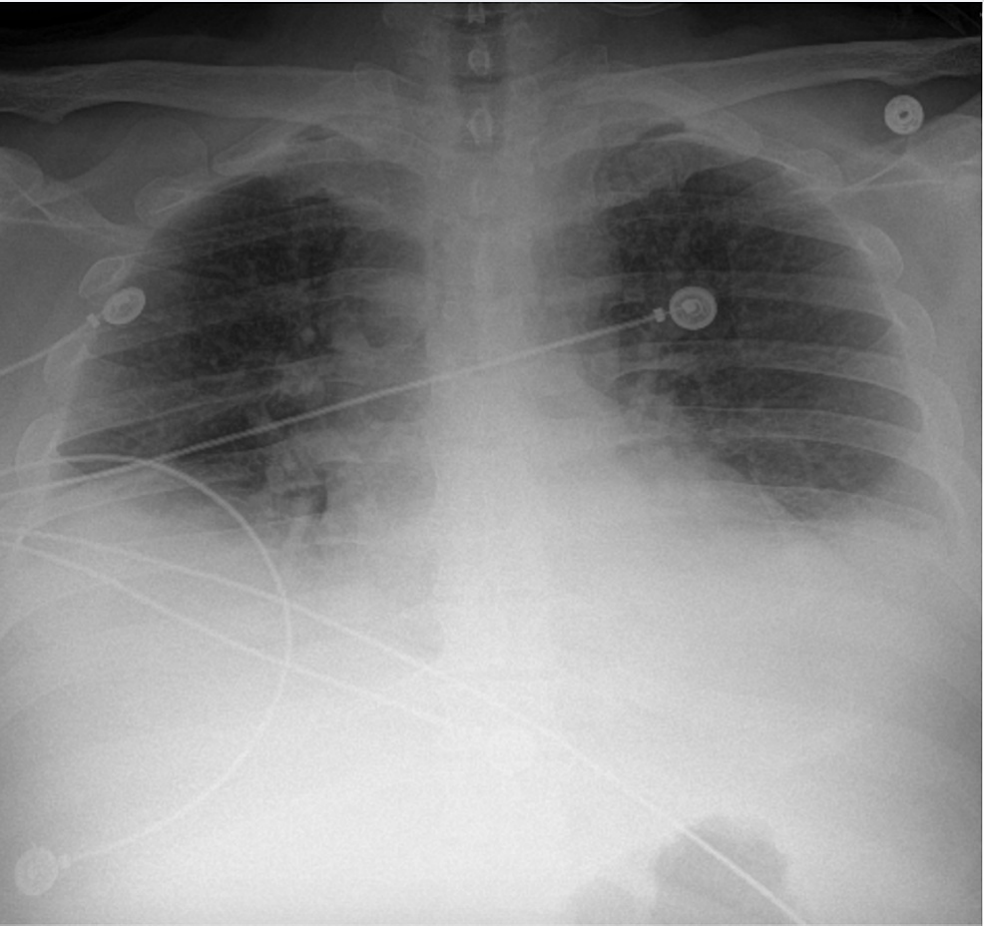
Chest X-ray showing bilateral infiltrates with blunting of the costophrenic angles.

The patient was admitted to the medical intensive care unit (ICU) for management of AP in the setting of elevated TGs. She was started on intravenous fluids and an insulin drip at a rate range of 0.1- 0.3 units/kg. She was allowed oral intake as tolerated. Her home medications including hormone replacement therapy medications, and estradiol and Aldactone were held. Once the TG levels were <500, she was taken off the insulin drip and transitioned to a subcutaneous insulin regimen over the next three days. Gemfibrozil and omega-3 fatty acid capsules were also added to the regimen. Her ICU course was complicated by the development of bilateral pleural effusions that responded to diuresis (Figure 3).

She was transferred to the general medical floor and was eventually discharged home and advised to take atorvastatin 40 mg daily, gemfibrozil 600 mg PO BID, and omega 3 fatty acid capsules. She is scheduled to follow up with an endocrinologist and primary care provider for further outpatient management.

## Discussion

Gallstones and alcohol abuse are the two most common causes of AP, with HTG responsible for only about 7% of the cases. The severity of pancreatitis increases as TG levels increase. Although the exact mechanism of hypertriglyceridemia-induced acute pancreatitis (HTG-AP) is poorly understood, it is thought to be related to high levels of free fatty acids (FFA). HTG with serum TG levels of ≥500 mg/dL (≥5.65 mmol/L) increases the risk of AP [2]. HTG is categorized into primary and secondary HTG based on the etiology. Primary HTG is induced by genetic or environmental factors. Secondary HTG can be due to uncontrolled diabetes mellitus, obesity, pregnancy, and medications, etc. Here, we focus on estrogen and hormone replacement therapies as the etiology of HTG and pancreatitis. Estrogen decreases the levels of hepatic lipase and, in turn, increases the levels of TG. The regulation of lipoprotein lipase (LPL) expressed in pancreatic islet cells is altered by estrogen as well. Estrogen promotes the synthesis of TG by the liver. In addition, estrogen is proposed to have direct toxic effects on the pancreatic cells suggested by the pancreatic amylase release in rats stimulated by estrogen [3]. The acinar cells of the exocrine pancreas produce lipase. Lipase breaks down TG to produce FFA and glycerol. With high levels of FFA, they aggregate into micelles. These micelles cause ischemia in the pancreas, trigger acidosis, which in turn activates lysosomal cathepsin-B and trypsinogen to form trypsin. FFA also has direct cytotoxic effects on the acinar and vascular endothelial cells causing endothelial dysregulation, vascular leakage, and coagulation cascade activation. HTG-induced microcirculatory disturbance leads to the release of thromboxane A2/PGI2 resulting in excessive contraction of the capillary bed and aggravation of the microcirculatory disturbance. HTG decreases the production of antioxidant products like superoxide dismutase and glutathione peroxidase. The imbalance of oxidative and antioxidative species initiates an inflammatory response and aggravates the pancreatic injury. The effect of estrogen on TG depends on the route of administration. Oral estrogens may raise the TG levels but transdermal forms have a null effect or a minimal impact on the levels. Topical estrogens used vaginally for postmenopausal women had minimal systemic absorption. The levels of estrogen in postmenopausal women using vaginal estrogens remained at postmenopausal levels, and thus had no effect on TG levels [4]. The clinical presentation of patients suffering from HTG-AP is similar to patients with AP from other etiologies. HTG-AP presents in a relatively younger population compared to AP from other causes. There is inconsistent data on the relationship between the level of TG and the severity of pancreatitis [1]. In a study of 121 patients evaluating the natural course of HTG-AP, local complications were higher in patients with TG ≥1,000 mg/dL, and chronic pancreatitis was reported in 17.8% of patients. Moreover, when compared with AP from other causes, the need for ICU admission (39% versus 16%; P ≤ 0.001), systemic inflammatory response syndrome (56% versus 28%; P ≤ 0.03), and persistent organ failure (23% versus 11%; P ≤ 0.05) were significantly higher in the HTG-AP cohort [5]. In a study of 144 patients with HTG-AP conducted between 1999 and 2013, higher TG levels were associated with worse outcomes. A receiver operating characteristic curve showed that a cut-off of TG level of 2,648 mg/dL was predictive of worse outcomes. The moderately severe and severe forms of HTG-AP were found in 50% of the patients in the low-TG group and 74.36% of the patients in the high-TG group (P = 0.004). Hospital length of stay, ICU length of stay, mortality, and recurrence rates at one year, which were the secondary endpoints in this study, were noted to be higher in the high-TG group, but this did not reach statistical significance [6]. When suspecting HTG-AP, amylase levels at the time of presentation can be normal due to colorimetric interference of lipemic serum. A repeat test should be performed after diluting serum. In addition, TG levels should be measured early at the time of presentation as the levels can reduce significantly with fasting.

Treatment

The cornerstone of the management of AP of any etiology is early recognition, aggressive hydration, pain management, and early enteral feeding. Scoring systems like Apache should be used for assessing the severity of pancreatitis. Severe pancreatitis should be managed in the ICU. Treatment of HTG-AP should focus on lowering the TG levels.

Insulin activates the LPL promoting the degradation of chylomicrons and lowering the TG levels. Insulin lowers the TG levels by 30-50%, which is often used in acute situations to rapidly lower the TG levels. Attention must be paid to potassium levels during such high doses of insulin infusion. Patients will likely need to be on dextrose infusion to allow insulin infusion without resultant hypoglycemia. Insulin infusion can be continued until the TG levels drop to less than 1,000 mg/dL. A meta-analysis of three studies with 118 patients concluded that insulin infusion has resulted in a statistically significant decrease in the length of hospitalization and Apache scores 72 hours after treatment [7].

On the other hand, heparin causes the release of LPL from endothelial cells. Concern for a rebound HTG with heparin infusion makes it less favorable for use in the treatment of HTG. With higher TG levels, aggressive and invasive interventions like PLEX and venovenous filtration are chosen. PLEX can rapidly lower the TG and inflammatory cytokine levels, thus downregulating the inflammatory process. One session lowers the TG levels by 50-80%. PLEX is effective but can be associated with adverse reactions. According to the World Apheresis Registry Report, the risk associated with PLEX is low at about 5.7%. PLEX also needs a central venous access which carries pertinent risks [1]. There is no high-quality data available, but typically PLEX is suggested as early as possible for patients with severe HTG-AP. The American Society for Apheresis considers HTG-AP as a Grade 2C recommendation for PLEX [1].

Once the patient can tolerate diet and the TG levels are below 1,000 mg/dL, medical therapy is initiated. Medical management is not effective at TG levels above 1,000 mg/dL as the drugs work primarily by reducing the synthesis and secretion by the liver. Treatment is usually with fenofibrates which lower the TG levels by 70%. Statins can be added to the treatment regimen. Statins typically lower the TG levels by 5-15% [8]. Weight loss, aerobic exercise, and avoidance of high glycemic index foods are suggested. Alcohol consumption should be avoided in patients with severe HTG. Omega-3 fatty acids have been shown to reduce the TG levels by up to 50% [9].

## Conclusions

HTG is associated with severe pancreatitis. Identification of HTG is an essential step in the treatment of AP. Drug-induced HTG is an important category, and hormone replacement therapy is an uncommon but important etiology, especially in the transgender population. Our case depicts the possibility of such a scenario occurring in the transgender population. Exacerbation of HTG, often with AP, and failure of TG-lowering treatment can result when women with overt or covert familial HTG are given hormone therapy. It shows the need for consideration of risk before starting estrogen therapy, especially with family history screening and consideration of monitoring TG in patients who have a first-degree relative with a history of HTG, diabetes mellitus, or AP.

## References

[1] Garg R, Rustagi T (2018). Management of hypertriglyceridemia induced acute pancreatitis. Biomed Res Int.

[2] Backes J, Anzalone D, Hilleman D, Catini J (2016). The clinical relevance of omega-3 fatty acids in the management of hypertriglyceridemia. Lipids Health Dis.

[3] Blevins GT Jr, Huang HS, Tangoku A, Mckay DW, Rayford PL (1991). Estrogens influence cholecystokinin stimulated pancreatic amylase release and acinar cell membrane cholecystokinin receptors in rat. Life Sci.

[4] Naessen T, Rodriguez-Macias K, Lithell H (2001). Serum lipid profile improved by ultra-low doses of 17 beta-estradiol in elderly women. J Clin Endocrinol Metab.

[5] Vipperla K, Somerville C, Furlan A (2017). Clinical Profile and Natural Course in a Large Cohort of Patients With Hypertriglyceridemia and Pancreatitis. J Clin Gastroenterol.

[6] Wang SH, Chou YC, Shangkuan WC, Wei KY, Pan YH, Lin HC (2016). Relationship between plasma triglyceride level and severity of hypertriglyceridemic pancreatitis. PLoS One.

[7] Li J, Chen TR, Gong HL, Wan MH, Chen GY, Tang WF (2012). Intensive insulin therapy in severe acute pancreatitis: a meta-analysis and systematic review. West Indian Med J.

[8] Chapman MJ, Ginsberg HN, Amarenco P (2011). Triglyceride-rich lipoproteins and high-density lipoprotein cholesterol in patients at high risk of cardiovascular disease: evidence and guidance for management. Eur Heart J.

[9] Skulas-Ray AC, Wilson PWF, Harris WS (2019). Omega-3 fatty acids for the management of hypertriglyceridemia: a science advisory from the American Heart Association. Circulation.

